# Supplementary material for: Psychological Distress and Food Insecurity among International Students at a Hungarian University: A Post-Pandemic Survey
Source: Nutrients. 2024 Jan 12;16(2):241. doi: 10.3390/nu16020241 (PMC10820830; doi:10.3390/nu16020241)
Supplement: Supplementary file 1 [file nutrients-16-00241-s001.zip › nutrients-2773654-supplementary.pdf]

Supplementary Table S1. Factors associated with psychological distress among the participants according to the bivariate analysis.

Variables	Depression		X	p	Anxiety		X	p	Stress		X	p
	No<br>n (%)	Yes<br>n (%)			No<br>n (%)	Yes<br>n (%)			No<br>n (%)	Yes<br>n (%)		
**Age (years)**			6.481	0.039			1.174	0.556			4.115	0.128
18-24	141 (61.3)	10 (64.3)			133 (64.9)	116 (60.1)			177 (63.0)	72 (61.5)		
25-29	54 (23.5)	48 (28.6)			48 (23.4)	54 (29.0)			66 (23.4)	36 (30.8)		
>=30	35 (15.2)	12 (7.1)			24 (11.7)	23 (12.0)			38 (13.5)	9 (7.7)		
**Gender**			1.331	0.249			7.079	0.008				
Male	112 (60.9)	72 (39.1)			108 (58.7)	76 (41.3)			143 (77.8)	41 (22.2)	8.345	0.004
Female	118 (55.1)	96 (44.9)			97 (45.3)	117 (54.7)			138 (64.4)	76 (35.6)		
**Region**			3.593	0.309			1.217	0.749			5.094	0.165
Asia	154 (61.1)	98 (38.9)			132 (52.4)	120 (47.6)			185 (73.4)	67 (26.6)		
Africa	54 (52.9)	48 (47.1)			52 (51.0)	50 (49.0)			71 (69.6)	31 (30.4)		
Europe	11 (45.8)	13 (54.2)			13 (54.2)	11 (45.8)			14 (58.3)	10 (41.7)		
Americas	11 (55.0)	9 (45.0)			8 (40.0)	12 (60.0)			11 (55.0)	9 (45.0)		
**Religious**			0.047	0.828			2.316	0.128			<0.001	0.997
No	50 (58.8)	35 (41.2)			50 (58.8)	35 (41.2)			60 (70.6)	25 (29.4)		
Yes	180 (57.5)	133 (42.5)			155 (49.5)	158 (50.5)			221 (70.6)	92 (29.4)		
**Relationship status**			5.354	0.021			0.677	0.411				
Single	152 (54.1)	129 (45.9)			141 (50.2)	140 (49.8)			189 (67.3)	92 (32.7)	5.148	0.023
Partnered	78 (66.7)	39 (33.3)			64 (54.7)	53 (45.3)			92 (78.6)	25 (21.4)		
**Living arrangements**			3.521	0.061			6.816	0.009			8.988	0.003
Alone	98 (63.6)	56 (36.4)			92 (59.7)	62 (40.3)			122 (79.2)	32 (20.8)		
Roommate (s)	82 (48.8)	78 (51.2)			72 (45.0)	88 (55.0)			98 (61.2)	62 (38.8)		
Family	50 (59.5)	34 (40.5)			41 (48.8)	43 (51.2)			61 (72.6)	23 (27.4)		
**Level of education**			1.456	0.228			0.666	0.414			2.898	0.089
Undergraduate	174 (59.6)	118 (40.4)			154 (52.7)	138 (47.3)			213 (72.9)	79 (27.1)		
Postgraduate	56 (52.8)	50 (47.2)			51 (48.1)	55 (51.9)			68 (64.2)	38 (35.8)		
**Field of study**			2.323	0.127			0.758	0.384			3.083	0.079
Healthcare	154 (60.6)	100 (39.4)			135 (53.1)	119 (46.9)			187 (73.6)	67 (26.4)		

Other	76 (52.8)	68 (47.2)			70 (48.6)	74 (51.4)			94 (65.3)	50 (34.7)		
**Mode of financing**			1.030	0.310			0.839	0.360			0.241	0.623
Scholarship	91 (54.8)	75 (45.2)			81 (48.8)	85 (51.2)			115 (69.3)	51 (30.7)		
Self-financing	139 (59.9)	93 (40.1)			124 (53.4)	108 (46.6)			166 (71.6)	66 (28.4)		
**Employment**			0.335	0.563			0.141	0.707			0.384	0.536
No	201 (57.3)	150 (42.7)			182 (51.9)	169 (48.1)			246 (70.1)	105 (29.9)		
Yes	29 (61.7)	18 (38.3)			23 (48.9)	24 (51.1)			35 (74.5)	12 (25.5)		
**Length of stay**			8.285	0.016			0.581	0.748			6.257	0.044
< 1	47 (48.0)	51 (52.0)			49 (50.0)	49 (50.0)			60 (61.2)	38 (38.8)		
1-4	80 (67.2)	39 (32.8)			59 (49.6)	60 (50.4)			91 (76.5)	28 (23.5)		
≥5	103 (56.9)	78 (43.1)			97 (53.6)	84 (46.4)			130 (71.8)	51 (28.2)		
**Language proficiency**			4.877	0.087			3.718	0.156			10.195	0.006
Poor	166 (54.8)	137 (45.2)			151 (49.8)	152 (50.2)			203 (67.0)	100 (33.0)		
Medium	49 (66.2)	25 (33.8)			39 (52.7)	35 (47.3)			58 (78.4)	16 (21.6)		
Good	15 (71.4)	6 (28.6)			15 (71.4)	6 (28.6)			20 (95.2)	1 (4.8)		
**Tobacco use**			2.449	0.118			1.952	0.162			2.062	0.151
No	203 (59.4)	139 (40.6)			181 (52.9)	161 (47.1)			246 (71.9)	96 (28.1)		
Yes	27 (48.2)	29 (51.8)			24 (42.9)	32 (57.1)			35 (62.5)	21 (37.5)		
**Alcohol consumption**			1.434	0.231			0.516	0.473			0.878	0.349
No/occasional consumption	199 (59.1)	138 (40.9)			171 (50.7)	166 (49.3)			241 (71.5)	96 (28.5)		
Regular consumption	31 (50.8)	30 (49.2)			34 (55.7)	27 (44.3)			40 (65.6)	21 (34.4)		
**Self-rated health**			5.345	0.021			7.801	0.005			16.722	<0.001
Good	170 (61.6)	106 (38.4)			155 (56.2)	121 (43.8)			212 (76.8)	64 (23.2)		
Poor	60 (49.2)	62 (50.8)			50 (41.0)	72 (59.0)			69 (56.6)	53 (43.4)		
**Food security status**			14.605	<0.001			17.435	<0.001			20.072	<0.001
Food secure	156 (65.5)	82 (34.5)			143 (60.1)	95 (39.9)			188 (79.0)	50 (21.0)		
Food insecure	74 (46.2)	86(53.8)			62 (38.7)	98 (61.3)			93 (85.1)	67 (41.9)		
